# Structural diversity and evolution of the N-terminal isoform-specific region of ecdysone receptor-A and -B1 isoforms in insects

**DOI:** 10.1186/1471-2148-10-40

**Published:** 2010-02-12

**Authors:** Takayuki Watanabe, Hideaki Takeuchi, Takeo Kubo

**Affiliations:** 1 Department of Biological Sciences, Graduate School of Science, The University of Tokyo, Bunkyo-ku, Tokyo 113-0033, Japan

## Abstract

**Background:**

The ecdysone receptor (EcR) regulates various cellular responses to ecdysteroids during insect development. Insects have multiple EcR isoforms with different N-terminal A/B domains that contain the isoform-specific activation function (AF)-1 region. Although distinct physiologic functions of the EcR isoforms have been characterized in higher holometabolous insects, they remain unclear in basal direct-developing insects, in which only A isoform has been identified. To examine the structural basis of the EcR isoform-specific AF-1 regions, we performed a comprehensive structural comparison of the isoform-specific region of the EcR-A and -B1 isoforms in insects.

**Results:**

The EcR isoforms were newly identified in 51 species of insects and non-insect arthropods, including direct-developing ametabolous and hemimetabolous insects. The comprehensive structural comparison revealed that the isoform-specific region of each EcR isoform contained evolutionally conserved microdomain structures and insect subgroup-specific structural modifications. The A isoform-specific region generally contained four conserved microdomains, including the SUMOylation motif and the nuclear localization signal, whereas the B1 isoform-specific region contained three conserved microdomains, including an acidic activator domain-like motif. In addition, the EcR-B1 isoform of holometabolous insects had a novel microdomain at the N-terminal end.

**Conclusions:**

Given that the nuclear receptor AF-1 is involved in cofactor recruitment and transcriptional regulation, the microdomain structures identified in the isoform-specific A/B domains might function as signature motifs and/or as targets for cofactor proteins that play essential roles in the EcR isoform-specific AF-1 regions. Moreover, the novel microdomain in the isoform-specific region of the holometabolous insect EcR-B1 isoform suggests that the holometabolous insect EcR-B1 acquired additional transcriptional regulation mechanisms.

## Backgrounds

Morphogenetic events during insect development are triggered by ecdysteroids. The major insect ecdysteroid, 20-hydroxyecdysone (20E), binds directly to a heterodimeric transcription factor comprising two nuclear receptors, the ecdysone receptor (EcR) and the ultraspiracle, to activate a complex transcriptional cascade [1]. In the holometabolous insects, there are two or three EcR isoforms (A and B1 isoforms; *Drosophila *has an additional B2 isoform) that are produced from a single genetic locus by differential promoter usage and alternative splicing [2]. The EcR isoforms have a common C-terminal region that includes the DNA binding domain and the ligand binding domain (the C-F domains), but also have isoform-specific regions in the N-terminal A/B domain. In the holometabolous insects, each EcR isoform govern distinct steroid-stimulated responses during metamorphosis. In *Drosophila*, the EcR-A isoform is predominantly expressed in the proliferative tissues during metamorphosis, and is required for adult-specific developmental processes. In contrast, the EcR-B isoforms (B1 and B2 isoforms) are expressed in the larva-specific tissues, and are involved in 20E-triggered larval tissue remodeling during metamorphosis [2-7]. In the red flour beetle Tribolium *castaneum*, the EcR-A isoform has a dominant role in transduction of the ecdysteroid action by regulating the expression of 20E-response genes [8]. Only the A isoform has been identified in basal direct-developing (ametabolous and hemimetabolous) insects [9,10], therefore, it is not known whether direct-developing insects have multiple EcR isoforms with distinct physiologic functions.

In addition to the differential expression patterns and the distinct physiologic functions during development, EcR isoforms have differential transcriptional regulation mechanisms. The A/B domain of the nuclear receptors generally contains the ligand-independent activation function (AF)-1 region [11]. Several studies of the AF-1 function of *Drosophila *EcR isoforms have revealed that the A/B domain of *Drosophila *EcR-B isoforms have strong transactivation activity, whereas that of the A isoform has weaker transactivation activity [12]. The AF-1 of *Drosophila *EcR-B1 mainly locates in the N-terminal region (amino acid residues 1-53), whose sequence is considerably conserved among higher holometabolous insects such as flies, mosquitoes, and moths [13]. Although structural differences in the AF-1 region among the EcR isoforms result in differential transcriptional regulation, the structural basis and molecular mechanisms underlying the isoform-specific AF-1 functions remain obscure.

In the present study, to examine the detailed structural characteristics of the isoform-specific region of each EcR isoform, we performed a comprehensive structural comparison of the isoform-specific regions of the insect EcR-A and -B1 isoforms. First, we newly identified EcR isoforms in 51 insect and non-insect arthropod species, focusing mainly on the basal direct-developing insects. Then, we compared the deduced amino acid sequences of the isoform-specific region of the EcR-A and -B1 isoforms (45 and 76 species, respectively). Sequence comparison revealed that the isoform-specific region of each EcR isoform contains several conserved microdomain structures, some of which were evolutionally acquired or modified. Interestingly, the isoform-specific region of the holometabolous insect EcR-B1 isoform contained a highly conserved (K/R)RRW motif at the N-terminus, which was lacking in basal direct-developing insects. Given that the nuclear receptor AF-1 regions provide an interaction interface for co-regulatory proteins, the conserved microdomain structures in the EcR isoform-specific AF-1 regions might play essential roles in transcriptional regulation. Moreover, the holometabolous insect EcR-B1 might have novel transcriptional regulation mechanisms that are mediated by the newly acquired (K/R)RRW motif.

## Results

### Cloning of cDNA fragments encoding the A/B domain of the EcR-A and -B1 isoforms

The EcR gene encodes two or three isoforms in the holometabolous insects (A, B1 and B2 isoforms), but only A isoform was previously identified in the direct-developing insects. In the present study, to perform a comprehensive sequence comparison of the isoform-specific region of the EcR-A and -B1 isoforms among insects and non-insect arthropods, we obtained cDNA fragments encoding the A/B domains of the EcR-A and -B1 isoforms from 18 and 51 arthropod species, respectively, by using reverse transcription polymerase chain reaction (see Additional file 1, Table S1 and Additional file 2, Table S2). We newly identified the EcR-A isoform in the direct-developing insects, including Paleoptera (1 species), Polyneoptera (2 species), and Paraneoptera (8 species). For a detailed analysis, we also identified the EcR-A isoform in 5 holometabolous insect species and one non-insect arthropod species. Although the EcR-B1 isoform was identified in many holometabolous insects and several non-insect arthropods, it has not been identified in basal hemimetabolous and ametabolous insects. Therefore, we newly identified the EcR-B1 isoform in basal insects, including Apterygota (2 species), Paleoptera (2 species), Polyneoptera (8 species), and Paraneoptera (12 species). In addition, we identified the EcR-B1 isoform in 25 holometabolous insect species and two non-insect arthropod species. To our knowledge, this is the first report of the identification of the EcR-B1 isoform in the direct-developing insects. In the direct-developing insects, the EcR isoforms were cloned from the whole body of the nymph and adult, and isolated tissues, including the nymphal wing pad, fat body, testis, and ovaries (data not shown), suggesting that the EcR isoforms are broadly distributed in the direct-developing insects.

### The A isoform-specific region was classified into five structural types

We compared the deduced amino acid sequences of the isoform-specific region in the A/B domain of the EcR-A isoform identified from 45 arthropod species (5 non-insect arthropod species [Crustacea and Chelicerata], 15 direct-developing insect species [1 paleopteran species, 4 polyneopteran species, and 9 paraneopteran species] and 26 Endopterygota species). GenBank accession numbers of the EcR-A isoforms were listed in Additional file 1, Table S1.

#### General structural features of the A isoform-specific region

The C-terminal region of the A isoform-specific region contained a conserved Ser/Thr-rich region, which was formally referred to as the "A-box" [10,14]. In the N-terminal region of the A isoform-specific region, we found two conserved microdomains: the SUMOylation motif and the nuclear localization signal (NLS). In addition, we found conserved (D/E)(D/E)W residues between the NLS and the C-terminal A-box. The SUMOylation motif in the A isoform-specific region contained a canonical consensus sequence for the SUMOylation (ΨKxE, where Ψ represents a large hydrophobic amino acid) [15]. The NLS of the A isoform-specific region contained a consensus sequence of the monopartite NLS [K(K/R)x(K/R)] [16].

Comprehensive structural comparison revealed several subgroup-specific structural modifications. Based on the structural similarity, we classified the isoform-specific region of the EcR-A isoform into five structural types.

#### Type-1 A isoform-specific region (Figure 1)

**Figure 1:**
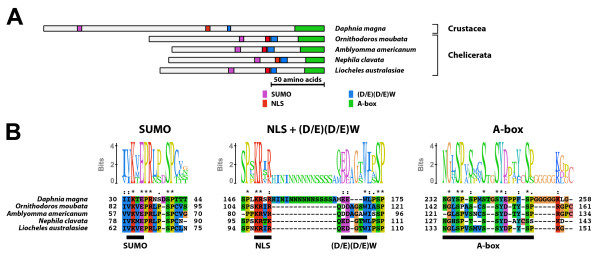
**Type-1 A isoform-specific region**. The type-1 A isoform-specific region contained four conserved microdomains. (A) Schematic representation of the type-1 A isoform-specific regions. Positions of the SUMOylation motif, the NLS, the (D/E)(D/E) residues, and the A-box are indicated by colored boxes. (B) The alignment and Sequence Logo representation of the conserved microdomains of the type-1 A isoform-specific region. The amino acid residues are represented in the default color scheme of ClustalX. Asterisks indicate identical residues and colons indicate similar residues.

The type-1 A isoform-specific region contained the SUMOylation motif, the NLS, the (D/E)(D/E)W residues, and the A-box. The consensus sequence of the A-box of the type-1 A isoform-specific region was NGxSPSxxSSYDxxYSP. The EcR-A isoform with the type-1 A isoform-specific region was identified in non-insect arthropods.

#### Type-2 A isoform-specific region (Figure 2)

**Figure 2:**
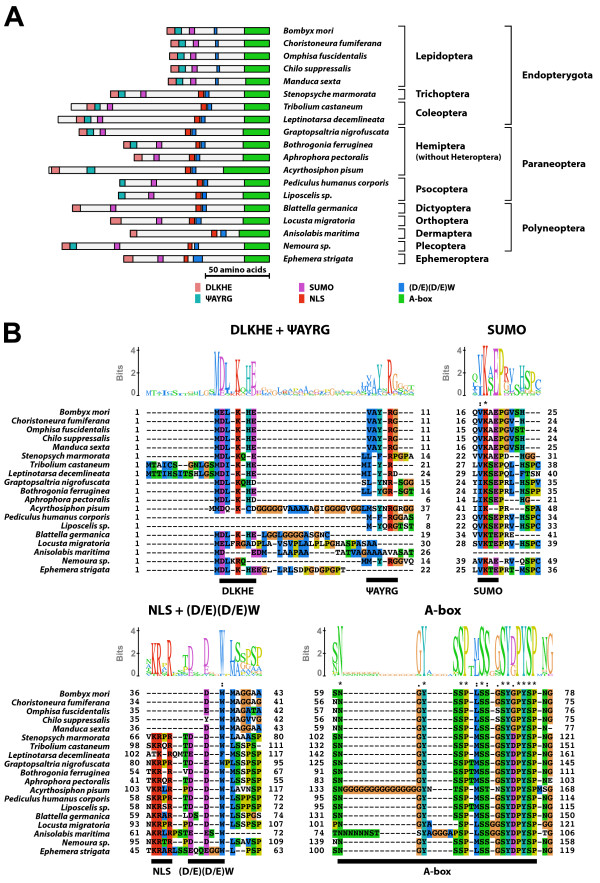
**Type-2 A isoform-specific region**. The type-2 A isoform-specific region contained four conserved microdomains and two conserved N-terminal sequences (DLKHE and ΨAYRG). (A) Schematic representation of the type-2 A isoform-specific regions. Positions of the two N-terminal sequences, the SUMOylation motif, the NLS, the (D/E)(D/E) residues, and the A-box are indicated by colored boxes. (B) The alignment and Sequence Logo representation of the N-terminal region and the conserved microdomains of the type-2 A isoform-specific region. The amino acid residues are represented in the default color scheme of ClustalX. Asterisks indicate identical residues and colons indicate similar residues.

The type-2 A isoform-specific region contained the SUMOylation motif, the NLS, the (D/E)(D/E)W residues, and the A-box. In addition, the type-2 A isoform-specific region generally contained two conserved N-terminal sequences (DLKHE and ΨAYRG, where Ψ represents a large hydrophobic amino acid). The consensus sequence of the A-box of the type-2 A isoform-specific region was NGYSSP(M/L)SSGSYDPYSP. The EcR-A isoform with the type-2 A isoform-specific region was identified in the direct-developing insects except for Heteroptera (Order Hemiptera) and three holometabolous orders (Coleoptera, Trichoptera, and Lepidoptera). The EcR-A isoforms identified from two psicopteran insects (*Pediculus humanus corporis *and *Liposcelis sp*.) did not contain the DLKHE sequence at their N-termini. The EcR-A isoforms identified from several polyneopteran insects (*Blattella germanica *[Order Dictyoptera], *Locusta migratoria *[Order Orthoptera], and *Anisolabis maritima *[Order Dermaptera]), and non-neopteran insects (Ephemera *strigata *[Order Ephemeroptera]) did not contain the ΨAYRG sequence at their N-termini. The EcR-A isoforms identified in Lepidoptera lacked the NLS.

#### Type-3 A isoform-specific region (Figure 3)

**Figure 3:**
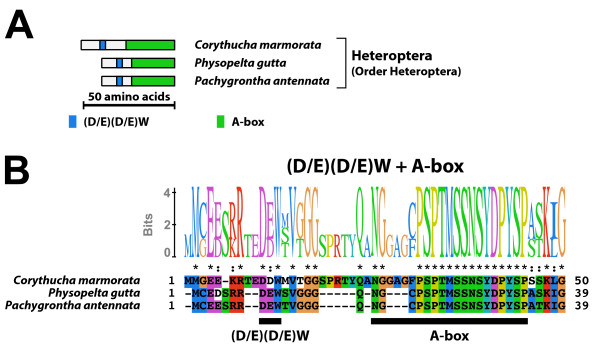
**Type-3 A isoform-specific region**. The type-3 A isoform-specific region contained two conserved microdomains. (A) Schematic representation of the type-3 A isoform-specific regions. Positions of the (D/E)(D/E) residues and the A-box are indicated by colored boxes. (B) The alignment and Sequence Logo representation of the full-length of the type-3 A isoform-specific regions. The amino acid residues are represented in the default color scheme of ClustalX. Asterisks indicate identical residues and colons indicate similar residues.

The type-3 A isoform-specific region contained the (D/E)(D/E)W residues and the A-box. The type-3 A isoform-specific region lacked the N-terminal region containing the SUMOylation motif and the NLS. The consensus sequence of the A-box of the type-3 A isoform-specific region was NGxPSPTMSSMSYDPYSP. The EcR-A isoform with the type-3 A isoform-specific region was identified in Heteroptera (Order Hemiptera).

#### Type-4 A isoform-specific region (Figure 4)

**Figure 4:**
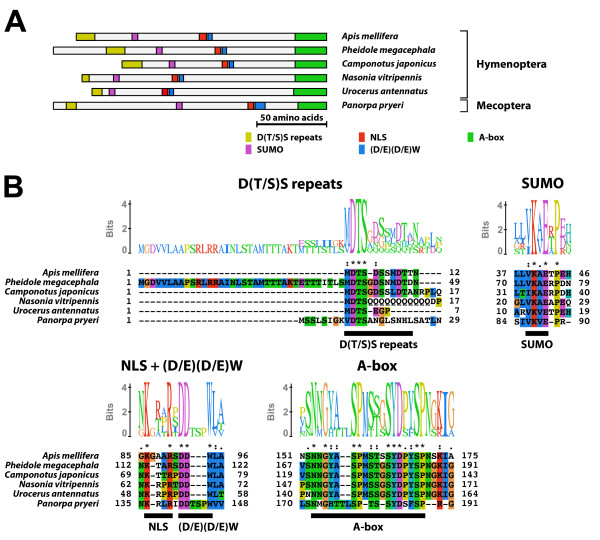
**Type-4 A isoform-specific region**. The type-4 A isoform-specific region contained four conserved microdomains and conserved N-terminal D(T/S)S repeats. (A) Schematic representation of the type-4 A isoform-specific regions. Positions of the D(T/S)S repeats, the SUMOylation motif, the NLS, the (D/E)(D/E) residues, and the A-box are indicated by colored boxes. (B) The alignment and Sequence Logo representation of the N-terminal region and the conserved microdomains of the type-4 A isoform-specific region. The amino acid residues are represented in the default color scheme of ClustalX. Asterisks indicate identical residues and colons indicate similar residues.

The type-4 A isoform-specific region contained the SUMOylation motif, the NLS, the (D/E)(D/E)W residues, and the A-box. In addition, the type-4 A isoform-specific region contained D(T/S)S repeats in the N-terminus. The consensus sequence of the A-box of the type-4 A isoform-specific region was NGYASPMS(T/S)GSYDPYSP. The EcR-A isoform with the type-4 A isoform-specific region was identified in Hymenoptera and Mecoptera. The NLS of the EcR-A isoforms identified from several hymenopteran insects (*Apis mellifera*, *Pheidole megacephala*, and *Camponotus japonicus*) was modified (consensus; KxxR).

#### Type-5 A isoform-specific region (Figure 5)

**Figure 5:**
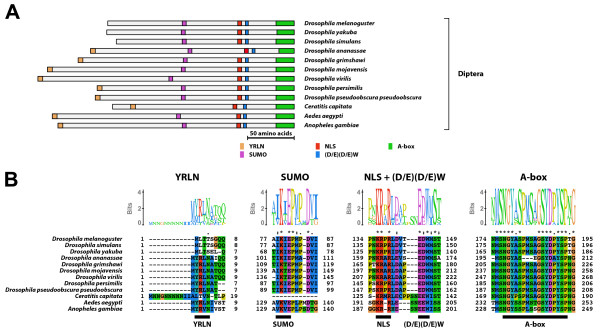
**Type-5 A isoform-specific region**. The type-5 A isoform-specific region contained four conserved microdomains and conserved N-terminal YRLN residues. (A) Schematic representation of the type-5 A isoform-specific regions. Positions of the YRLN residues, the SUMOylation motif, the NLS, the (D/E)(D/E) residues, and the A-box are indicated by colored boxes. (B) The alignment and Sequence Logo representation of the N-terminal region and the conserved microdomains of the type-5 A isoform-specific region. The amino acid residues are represented in the default color scheme of ClustalX. Asterisks indicate identical residues and colons indicate similar residues.

The type-5 A isoform-specific region contained the SUMOylation motif, the NLS, the (D/E)(D/E)W residues, and the A-box. In addition, the type-5 A isoform-specific region contained YRLN residues at the N-terminus. The consensus sequence of the A-box of the type-5 A isoform-specific region was NGYASPMSAGSYDPYSPNG. The EcR-A isoform with the type-5 A isoform-specific region was identified in Diptera. The EcR-A isoforms identified from several species in genus *Drosophila *(*D. melanogaster, D. simulans*, and *D. yakuba*) did not contain the YRLN sequence at their N-termini.

### The B1 isoform-specific region was classified into six structural types

We compared the deduced amino acid sequences of the isoform-specific region in the A/B domain of the EcR-B1 isoforms identified from 76 arthropod species (4 non-insect arthropod species [Crustacea, Myriapoda, and Chelicerata], 24 direct-developing insect species [2 apteryogote species, 2 Paleopteran species, 8 polyneopteran species, and 12 paraneopteran species] and 48 Endopterygota species). GenBank accession numbers of the EcR-B1 isoforms were listed in Additional file 2, Table S2.

#### General structural features of the B1 isoform-specific region

The N-terminal region of the B1 isoform-specific region essentially contained three microdomains: the S-rich motif, the SP residues, and the DL-rich motif. The S-rich motif was composed of acidic residues (D and E) and neutral polar residues (S and T), and contained a conserved core EV(T/S)SS sequence. The DL-rich motif was composed of repeats of acidic residues and aromatic/bulky hydrophobic residues (W, A, F, I, L, M, and V). The region between the S-rich and the DL-rich motifs generally contains conserved SP residues. In addition to these microdomains, the isoform-specific region of the holometabolous insect EcR-B1 isoform contained an additional conserved (K/R)RRW motif at the N-terminus.

Comprehensive structural comparison revealed several subgroup-specific structural modifications. Based on the structural similarity, we classified the isoform-specific region of the EcR-B1 isoform into seven structural types.

#### Type-1 B1 isoform-specific region (Figure 6)

**Figure 6:**
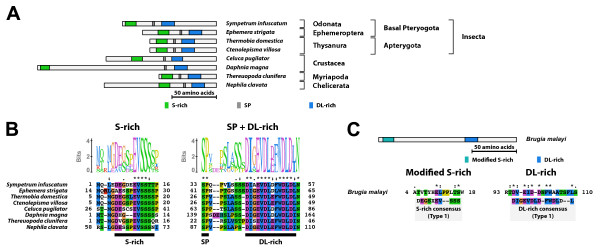
**Type-1 B1 isoform-specific region**. The type-1 B1 isoform-specific region contains three conserved microdomains. (A) Schematic representation of the type-1 B1 isoform-specific regions. Positions of the S-rich motif, the SP residues, and the DL-rich motif are indicated by colored boxes. (B) The alignment and Sequence Logo representation of the conserved microdomains found in the type-1 B1 isoform-specific region. The amino acid residues are represented in the default color scheme of ClustalX. Asterisks indicate identical residues and colons indicate similar residues. (C) The N-terminal region of *Brugia malayi *EcR contained the S-rich motif-like sequence (modified S-rich motif) and the DL-rich motif. Positions of the modified S-rich motif and the DL-rich motif are indicated in the schematic representation of the N-terminal region of *B. malayi *EcR (amino acid residues 1-151). The alignment of the microdomains found in *B. malayi *with their corresponding microdomains in the type-1 B1 isoform-specific region reveals their sequence similarity. The amino acid residues are represented in the default color scheme of ClustalX. Asterisks indicate identical residues and colons indicate similar residues.

The type-1 B1 isoform-specific region contained three conserved microdomains (the S-rich motif, the SP residues, and the DL-rich motif). The consensus sequences of the S-rich motif and the DL-rich motif of the type-1 B1 isoform-specific region were GDExSxEVSSSS and DIGEVDLDFWDLDL, respectively. The N-terminal region and the region between each two conserved microdomains varied in length and sequence. The EcR-B1 isoform with the type-1 B1 isoform-specific region was identified in non-insect arthropods and non-neopteran insects.

Moreover, the EcR isoforms identified from the filarial nematode *Brugia malayi *[EcR-A and -C isoforms (GenBank accession numbers; ABQ28713 and ABQ28714)] contained the S-rich motif-like sequence and the DL-rich motif in the N-terminal A/B domain (amino acid residues 1-153, both isoforms contained the common A/B domain; Figure 6C).

#### Type-2 B1 isoform-specific region (Figure 7)

**Figure 7:**
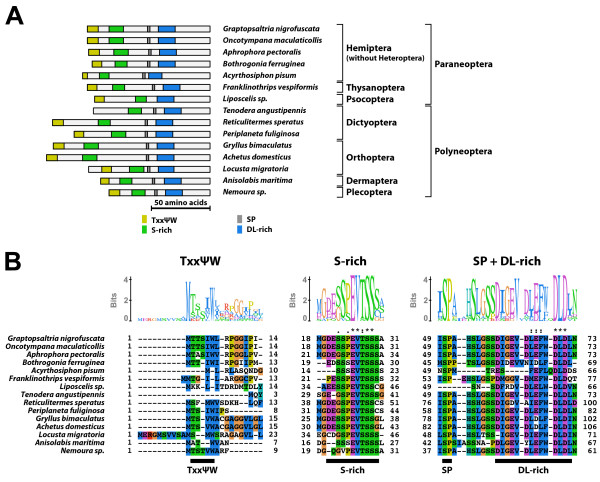
**Type-2 B1 isoform-specific region**. The type-2 B1 isoform-specific region contained three conserved microdomains and the N-terminal TxxΨW sequence. (A) Schematic representation of the type-2 B1 isoform-specific regions. Positions of the TxxΨW sequence, the S-rich motif, the SP residues, and the DL-rich motif are indicated by colored boxes. (B) The alignment and Sequence Logo representation of the N-terminal region and the conserved microdomains of the type-2 B1 isoform-specific region. The amino acid residues are represented in the default color scheme of ClustalX. Asterisks indicate identical residues and colons indicate similar residues.

The type-2 B1 isoform-specific region contained three conserved microdomains (the S-rich motif, the SP residues, and the DL-rich motif). In addition, the type-2 B1 isoform-specific region generally contained a partially conserved TxxΨW sequence at the N-terminus. The consensus sequences of the S-rich motif and the DL-rich motif of the type-2 B1 isoform-specific region were GxESSPEVTSSS and DIGEVDLEFWDLDL, respectively. The EcR-B1 isoform with the type-2 B1 isoform-specific region was identified in Polyneoptera and Paraneoptera except for Heteroptera (Order Hemiptera).

#### Type-2' B1 isoform-specific region (Figure 8)

**Figure 8:**
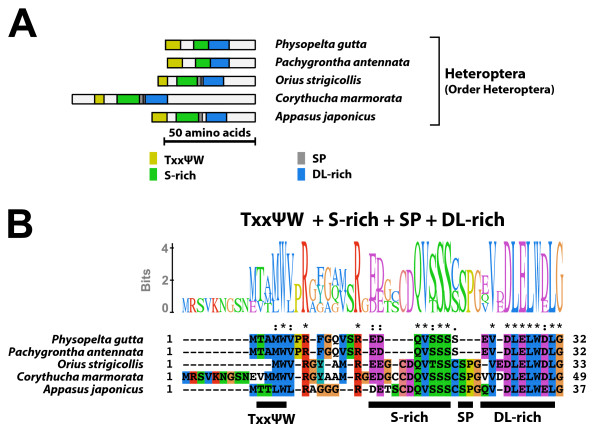
**Type-2' B1 isoform-specific region**. The type-2' B1 isoform-specific region contained three conserved microdomains and the N-terminal TxxΨW sequence. (A) Schematic representation of the type-2' B1 isoform-specific regions. Positions of the TxxΨW sequence, the S-rich motif, the SP residues, and the DL-rich motif are indicated by colored boxes. (B) The alignment and Sequence Logo representation of the N-terminal region of the type-2' B1 isoform-specific region. The amino acid residues are represented in the default color scheme of ClustalX. Asterisks indicate identical residues and colons indicate similar residues.

The type-2' B1 isoform-specific region structurally resembled to the type-2 B1 isoform-specific region, but the motif structures were modified. The consensus sequences of the S-rich motif and the DL-rich motif of the type-2' B1 isoform-specific region were EDxxxQVSSS and EVDLELWDLGL, respectively. Moreover, the region between the S-rich motif and the DL-rich motif were shortened. In some cases, these two motifs were completely fused (*e.g*. EcR-B1 isoforms of *Physopelta gutta *and *Pachygrontha antennata*). Like the type-2 B1 isoform-specific region, the type-2' B1 isoform-specific region contained the N-terminal TxxΨW sequence. The EcR-B1 isoform with the type-2' B1 isoform-specific region was identified in Heteroptera (Order Hemiptera).

#### Type-3 B1 isoform-specific region (Figure 9)

**Figure 9:**
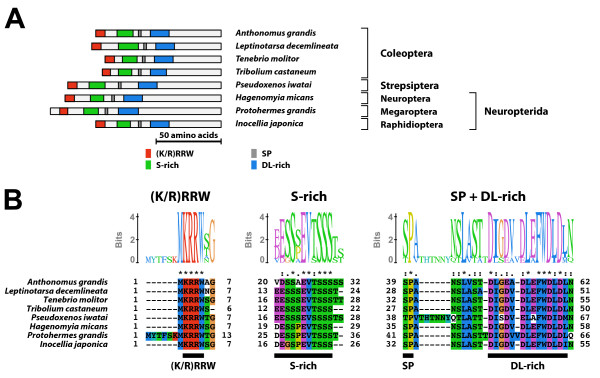
**Type-3 B1 isoform-specific region**. The type-4 B1 isoform-specific region contained four conserved microdomains. (A) Schematic representation of the type-4 B1 isoform-specific regions. Positions of the (K/R)RRW motif, the S-rich motif, the SP residues, and the DL-rich motif are indicated by colored boxes. (B) The alignment and Sequence Logo representation of the conserved microdomains of the type-4 B1 isoform-specific region. The amino acid residues are represented in the default color scheme of ClustalX. Asterisks indicate identical residues and colons indicate similar residues.

The type-3 B1 isoform-specific region contained four conserved microdomains [the (K/R)RRW motif, the S-rich motif, the SP residues, and the DL-rich motif]. The consensus sequences of the S-rich motif and the DL-rich motif of the type-3 B1 isoform-specific region were EESSSEVTSSS and DIGDVDLEFWDLDL, respectively. The EcR-B1 isoform with the type-3 B1 isoform-specific region was identified in Coleoptera, Strepsiptera, Neuroptera, Megaroptera, and Raphidioptera.

#### Type-4 B1 isoform-specific region (Figure 10)

**Figure 10:**
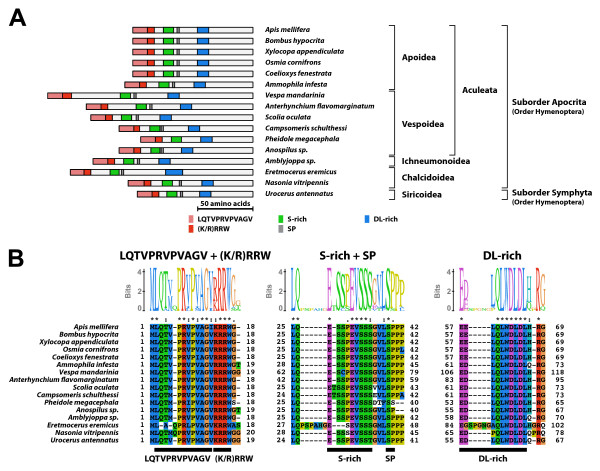
**Type-4 B1 isoform-specific region**. The type-4 B1 isoform-specific region contained four conserved microdomains and the N-terminal LQTVPRVPVAGV sequence. (A) Schematic representation of the type-4 B1 isoform-specific regions. Positions of the LQTVPRVPVAGV sequence, the (K/R)RRW motif, the S-rich motif, the SP residues, and the DL-rich motif are indicated by colored boxes. (B) The alignment and Sequence Logo representation of the N-terminal region and the conserved microdomains of the type-4 B1 isoform-specific region. The amino acid residues are represented in the default color scheme of ClustalX. Asterisks indicate identical residues and colons indicate similar residues.

The type-4 B1 isoform-specific region contained four conserved microdomains [the (K/R)RRW motif, the S-rich motif, the SP residues, and the DL-rich motif]. In addition, the type-4 B1 isoform-specific region contained a conserved LQTVPRVPVAGV sequence just N-terminally adjacent to the (K/R)RRW motif. The consensus sequences of the S-rich motif and the DL-rich motif of the type-4 B1 isoform-specific region were ESSPEVSSS and EDLQLWDLDL, respectively. The EcR-B1 isoform with the type-4 B1 isoform-specific region was identified in the aculeate Hymenoptera and horntails (Family Siricidae, Order Hymenoptera). The EcR-B1 isoform identified from two vespine wasps (*Vespa mandarinia *and *Anterhynchium flavomarginatum*) contained a S/H-rich insertion between the (K/R)RRW and the S-rich motifs.

#### Type-5 B1 isoform-specific region (Figure 11)

**Figure 11:**
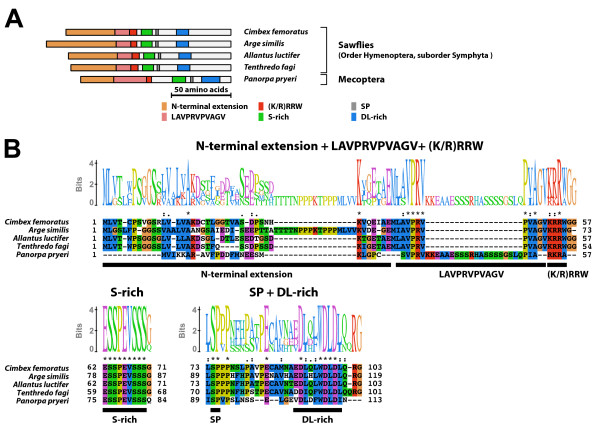
**Type-5 B1 isoform-specific region**. The type-5 B1 isoform-specific region contained four conserved microdomains, the N-terminal LAVPRVPVAGV sequence, and the N-terminal extension region. (A) Schematic representation of the type-5 B1 isoform-specific regions. Positions of the N-terminal extension, the LAVPRVPVAGV sequence, the (K/R)RRW motif, the S-rich motif, the SP residues, and the DL-rich motif are indicated by colored boxes. (B) The alignment and Sequence Logo representation of the N-terminal region and the conserved microdomains of the type-5 B1 isoform-specific region. The amino acid residues are represented in the default color scheme of ClustalX. Asterisks indicate identical residues and colons indicate similar residues.

The type-5 B1 isoform-specific region structurally resembled to the type-4 B1 isoform-specific region, but contained an additional N-terminal extension region. The consensus sequences of the conserved N-terminal sequence, the S-rich motif and the DL-rich motif of the type-5 B1 isoform-specific region were LAVPRVPVAGV, ESSPEVSSS, and EDLQLWDLDL, respectively. The EcR-B1 isoform with the type-5 B1 isoform-specific region was identified in sawflies, the basal lineage of Hymenoptera. Moreover, the isoform-specific region of the EcR-B1 isoform identified in *Panorpa pryeri *(Order Mecoptera) contained an N-terminal extension region similar to that of the type-5 B1 isoform-specific region. The *P. pryeri *EcR-B1 isoform contained the S-rich motif (amino acid residues 75-83; ESSPEVSSS) and the DL-rich motif (amino acid residues 99-112; ELGEVDLDFWDLDI), which were structurally similar to the S-rich motif of the type-4 B1 isoform-specific region and the DL-rich motif of type-3 B1 isoform-specific region, respectively.

#### Type-6 B1 isoform-specific region (Figure 12)

**Figure 12:**
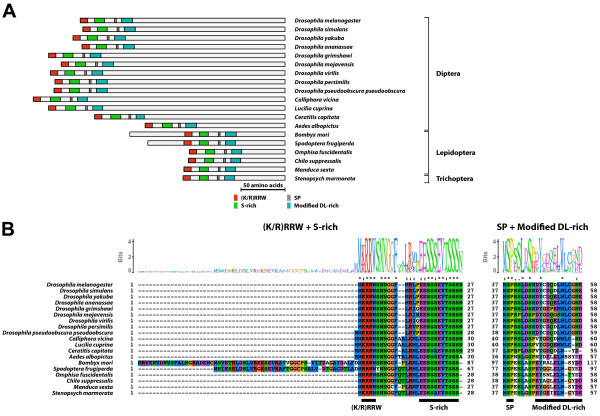
**Type-6 B1 isoform-specific region**. The type-6 B1 isoform-specific region contained four conserved microdomains. The DL-rich motif was structurally modified in the type-6 B1 isoform-specific region (termed as modified DL-rich motif). (A) Schematic representation of the type-6 B1 isoform-specific regions. Positions of the (K/R)RRW motif, the S-rich motif, the SP residues, and the modified DL-rich motif are indicated by colored boxes. (B) The alignment and Sequence Logo representation of the conserved microdomains of the type-6 B1 isoform-specific region. The amino acid residues are represented in the default color scheme of ClustalX. Asterisks indicate identical residues and colons indicate similar residues.

The type-6 B1 isoform-specific region contained conserved microdomains [the (K/R)RRW motif, the S-rich motif, the SP residues, and the DL-rich motif]. The consensus sequence of the S-rich motif of the type-6 B1 isoform-specific region was EESSSEVTSSS. Although the DL-rich motif of type-6 B1 isoform-specific region was composed of acidic residues and bulky hydrophobic residues, its sequence was highly modified [consensus; (D/E)Y(C/G)(E/D)LWxxxxD)]. The EcR-B1 isoform with the type-6 B1 isoform-specific region was identified in Diptera, Lepidoptera, and Trichoptera.

### Phylogenetic evolution of motif structures in the EcR isoform-specific regions in insects

**Figure 13:**
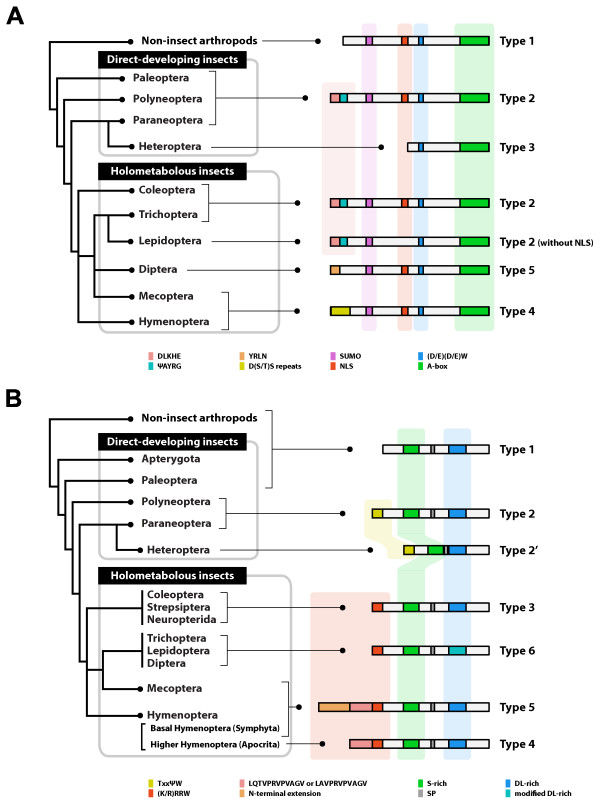
**Structural diversity of the EcR isoform-specific region in insects**. (A) Evolution model for the structural modification in the A isoform-specific region in insects. (B) Evolution model for the structural modification in the B1 isoform-specific region in insects. Schematic representation shows the phylogenetic relationship of insect subgroups with the structural types of the isoform-specific region. Conserved microdomains are indicated by colored boxes.

Based on the phylogenetic relationship of insect orders, we constructed evolution models for the structural evolution of the isoform-specific region of the EcR isoforms in insects (Figure 13). The structural modifications in the A isoform-specific region accompanied by insect evolution are represented in Figure 13A. The A isoform-specific region of non-insect arthropods (type-1 A isoform-specific region) was composed of four conserved microdomains [the SUMOylation motif, the NLS, the (D/E)(D/E)W residues, and the A-box]. The EcR-A isoform of direct developing insects acquired two N-terminal sequences (DLKHE and ΨAYRG) (type-2 A isoform-specific region), which were conserved among most direct-developing insect groups except for Heteroptera, whose EcR-A isoform lacked the N-terminal half of the isoform-specific region including the SUMOylation motif and the NLS (type-3 A isoform-specific region). The two N-terminal sequences and four conserved microdomains were also conserved among most holometabolous insect groups except for Hymenoptera, Mecoptera and Diptera. The isoform-specific region of the hymenopteran/mecopteran EcR-A isoform and the dipteran EcR-A isoform lacked the two N-terminal sequences, but contained the subgroup-specific sequences: The A isoform-specific region of the hymenopteran/mecopteran EcR-A isoform (type-4 A isoform-specific region) contained the D(S/T)S repeats, and that of the dipteran EcR-A isoform generally contained the YRLN sequence at the N-terminus. In addition, although the isoform-specific region of the lepidopteran EcR-A isoform structurally classified as the type-2 A isoform-specific region, it lacked the NLS.

The structural modifications in the B1 isoform-specific region that accompanies insect evolution are represented in Figure 13B. All types of the B1 isoform-specific region contained three conserved microdomains: the S-rich motif, the SP residues, and the DL-rich motif (the B1 isoform-specific region of several heteropteran insects did not contain the SP residues). The DL-rich motif of the type-6 B1 isoform-specific region was structurally modified and showed a limited sequence similarity with the corresponding motif of other insects. Interestingly, although Mecoptera is thought to be a sister taxon of Diptera [17], the *P. pryeri *EcR-B1 isoform had a DL-rich motif quite similar to the corresponding motif of the type-3 B1 isoform-specific region.

In addition to the three conserved microdomains, the isoform-specific region of the EcR-B1 isoform contained subgroup-specific N-terminal sequences. The EcR-B1 isoform of polyneopteran and paraneopteran insects (types 2 and 2' B1 isoform-specific region) generally contained the N-terminal TxxΨW sequence. In the holometabolous insects, the TxxΨW sequence was replaced with the (K/R)RRW motif, a novel conserved microdomain at the N-terminal end of the holometabolous insect EcR-B1 (types 3-6 B1 isoform-specific region). The hymenopteran/mecopteran EcR-B1 isoform contained the N-terminal LQTVPRVPVAGV (or LAVPRVPVAGV) sequence just N-terminally adjacent to the (K/R)RRW motif (types 4 and 5 B1 isoform-specific region). Moreover, the EcR-B1 isoform of the basal hymenopteran insects (sawflies) and the mecopteran insect had the N-terminal extension region (type 5 B1 isoform-specific region).

## Discussion

Generally, the nuclear receptor A/B domain contains the ligand-independent AF-1 transactivation domain that is involved in the interaction with transcriptional cofactors [18,19], and some nuclear receptors have multiple isoforms with different A/B domains that exhibit isoform-specific transcriptional regulation and physiological functions [20-23]. In the present study, we performed a structural analysis of the isoform-specific A/B domain of the EcR-A and -B1 isoforms. Our data revealed the presence of the conserved microdomain structures as well as the insect subgroup-specific structural variations in the isoform-specific region in the A/B domain of each EcR isoform. The conserved microdomain structures in the EcR isoform-specific regions might be the fundamental structural basis for the EcR isoform-specific AF-1 functions.

The A isoform-specific region generally contained the SUMOylation motif, the NLS, the (D/E)(D/E)W residues, and the A-box. Among these structural motifs, the (D/E)(D/E)W residues and the A-box were highly conserved in the C-terminal half of the A isoform-specific region across arthropod species, suggesting that these two microdomains are the essential structures for the A isoform-specific AF-1. Moreover, the NLS was conserved among all species except for Heteroptera and Lepidoptera. The NLS in the isoform-specific region of *Drosophila *EcR-A isoform is involved in nucleo-cytoplasmic shuttling in mammalian cells [24]. Our data suggest that the isoform-specific region-mediated nuclear localization mechanism of the EcR-A isoform is conserved among most species.

The A/B domain of *Drosophila *EcR-A isoform exhibits weak transactivation activity in *Drosophila *Kc167 cells [12], whereas it exhibits transcriptional inhibition activity in HeLa cells [13]. In the red flour beetle *T. castaneum*, the EcR-A isoform initiates ecdysone responses by regulating the expression of downstream 20E-response genes, indicating that the EcR-A isoform functions as a transactivating factor. In the N-terminal half of the A isoform-specific region, we found a conserved SUMOylation motif. SUMOylation is a reversible post-translational modification involved in various cellular processes, such as nucleo-cytoplasmic shuttling and transcriptional regulation [25-27]. SUMOylation of transcription factors generally results in the repression of transcriptional activation activity [28]. The high level of conservation of the SUMOylation motif in the A isoform-specific region suggests that it is functionally important. SUMOylation of the A isoform-specific region might contribute to the cell-type/organism-dependent transcriptional regulation mediated by the A/B domain of the EcR-A isoform.

All types of the B1 isoform-specific region contained the S-rich and the DL-rich conserved motifs. The isoform-specific region of the holometabolous insect EcR-B1 isoform (types 3-6 B1 isoform-specific region) contained an additional (K/R)RRW motif at the N-terminus. In *Drosophila*, strong transactivation activity was mapped at the N-terminal end of the A/B domain of the EcR-B1 isoform (amino acid residues 1-53) [13], which includes all three conserved motifs. Moreover, the S-rich motif-like sequences and the DL-rich motif are found in the A/B domain of the ecdysone receptor identified from the filarial nematode *B. malayi*. The high level of conservation of the S-rich and DL-rich motifs across ecdysozoan species suggests that these motifs are the essential structures for the B1 isoform-specific AF-1 function.

The length and sequences of the linker regions between microdomains, as well as the distribution of microdomains in the isoform-specific region varied across species. For example, the B1 isoform-specific region of the non-insect arthropod EcR-B1 isoforms (type-1 B1 isoform-specific region) contained a long linker region between the S-rich and the DL-rich motifs (e.g., *Daphnia magna *EcR-B1 contained an 137-amino acid linker region). In the holometabolous insects, the three conserved motifs [the (K/R)RRW, the S-rich, and the DL-rich motifs] were contained in the N-terminal half of the B1 isoform-specific region, and the linker regions between the motifs were shortened (e.g. type-6 B1 isoform-specific region). The isoform-specific region of the EcR-B1 isoform identified in vespoid wasps contained a long S/H-rich insertion between the holometabolous insect-specific (K/R)RRW motif and the S-rich motif. These data suggest that the conserved microdomains in the EcR isoform-specific regions are structurally, and even functionally, independent of each other.

*In vitro *and *in silico *structural analysis for the A/B domain of the *Drosophila *EcR isoforms revealed that the N-terminal region of the *Drosophila *EcR-B1 isoform-specific region, which contains the conserved microdomains, is structurally disordered [29]. The intrinsically disordered AF-1 region was found in many other nuclear receptors, such as the androgen receptor and the glucocorticoid receptor [30-34]. The intrinsic disordered regions are found in a lot of proteins involved in protein-protein interactions [35,36] and that the nuclear receptor AF-1 region is involved in cofactor recruitment and transcriptional regulation. Thus, the conserved microdomains found in the EcR isoform-specific region might function as signature motifs and/or target sites for the EcR isoform-specific transcription cofactors.

**Figure 14:**
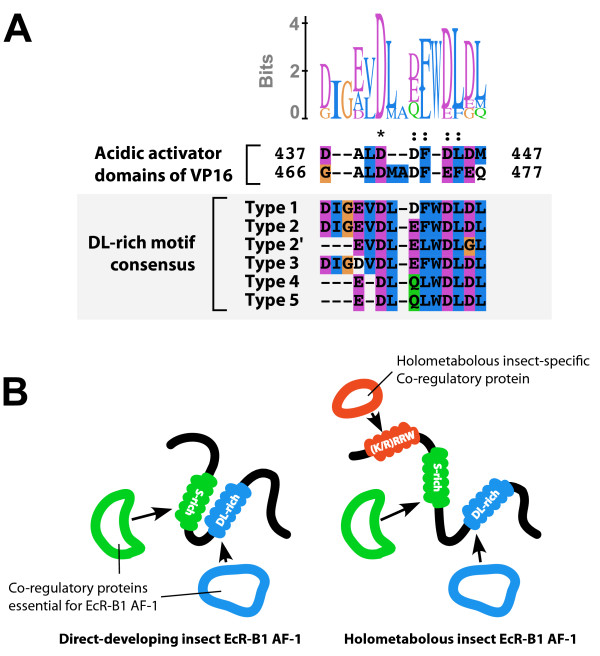
**Hypothetical model of the B1 isoform-specific region mediated transcriptional regulation**. (A) Sequence comparison between two acidic activator domains of herpes virus protein VP16 and the consensus sequences of the DL-rich motif of the EcR-B1 isoform. Two acidic activation domains of VP16 (GenBank accession number; NP_044650) were aligned with the consensus sequences of the DL-rich motifs of the type 1-5 B1 isoform-specific regions. The DL-rich motif of EcR-B1 isoform and the VP16 acidic activation domains comprise tandem repeats of acidic residues and aromatic/bulky hydrophobic residues. The amino acid residues are represented in the default color scheme of ClustalX. Asterisks indicate identical residues and colons indicate similar residues. (B) Schematic model of the action of the EcR-B1 isoform-specific AF-1. The S-rich and DL-rich motifs might interact with co-regulatory proteins essential for B1 isoform-specific AF-1. The holometabolous insect EcR-B1 isoform might have acquired a novel interaction partner(s) in the holometabolous insect-specific (K/R)RRW motif.

The A/B domain of the *Drosophila *EcR-B1 isoform activates transcription even in yeast and mammalian cells [13,37,38], therefore it is likely that the AF-1 region of the EcR-B1 isoform activates transcription through mechanisms conserved among eukaryotic organisms. Among the conserved microdomains contained in the *Drosophila *EcR-B1 isoform-specific region, the DL-rich motif, which comprises tandem repeats of acidic residues (D and E) and aromatic/bulky hydrophobic residues (W, A, F, I, L, M, and V), was structurally similar to the acidic activation domain (Figure 14A). Acidic activation domains are found in viral and cellular transcription factors such as VP16, GAL4 and p53 [39-42], as well as in the AF-1 region of several nuclear receptors [43,44]. Further studies, such as protein-protein interaction studies, will shed light on the transcriptional regulation mechanism of the EcR-B1 isoform-specific AF-1.

In the present study, we discovered that the N-termini of holometabolous insect EcR-B1 isoforms contain a novel conserved (K/R)RRW motif, which is lacking in the direct-developing insects. The (K/R)RRW motif was found in the N-terminal region of the EcR-B1 isoform identified in Hymenoptera, the most basal lineage of holometabolous insects, suggesting that the (K/R)RRW motif was acquired in association with the evolution of holometabolous insects. Strikingly high conservation of the (K/R)RRW motif across holometabolous insect species suggests its importance in the AF-1 function of the holometabolous insect EcR-B1 isoform. Taken together, we hypothesize that the holometabolous insect EcR-B1 isoform acquired a novel transcriptional regulation mechanism that is mediated by the (K/R)RRW motif (Figure 14B).

## Conclusions

A comprehensive structural comparison of the N-terminal isoform-specific region in the A/B domain of the EcR-A and -B1 isoforms in insects revealed several microdomain structures that are conserved across insects or acquired/modified in a specific-subgroup of insects. These microdomains might be the essential structural basis for the EcR isoform-specific AF-1 transactivation functions by serving as binding sites for co-regulatory proteins or as post-translational modification sites. The novel (K/R)RRW motif in the isoform-specific region of the holometabolous insect EcR-B1 isoform might provide additional interaction sites for co-regulatory proteins and mediate the holometabolous insect-specific regulation of the B1 isoform-specific AF-1 transactivation function. Identification of binding partners of the EcR isoform-specific regions and structural analysis of the interaction interface will provide further insight into the evolutionary importance of the structural modifications of the EcR isoform-specific AF-1 regions.

## Methods

### Animals

We collected 51 species of insects and non-insect arthropods for EcR cDNA cloning. Some of them were collected in the field and others were purchased. Their collection sites are listed in Additional file 3, Table S3. *Franklinothrips vespiformis, Orius strigicollis *and *Eretmocerus eremicus *were purchased from Arysta LifeScience (Tokyo, Japan). Colonies of *Apis mellifera *were purchased from Kumagaya bee farm (Saitama, Japan). Pupae of *Osmia cornifrons *were purchased from Mason Bee Research Institute (Sendai, Miyagi, Japan). Pupae of *Nasonia vitripennis *were purchased from Nosan corporation (Tsukuba, Ibaraki, Japan). Gryllus bimaculatus, *Acheta domestica *and *Thermobia domestica *were purchased from local pet stores.

### RNA isolation and reverse transcription

Tissues were dissected in ice-cold phosphate-buffered saline (138 mM NaCl, 3 mM Na_2_HPO_4_, 10 mM NaH_2_PO_4_, pH 7.4), and immediately homogenized in TRIzol reagent (Invitrogen, Carlsbad, CA) or stored in RNA *later *reagent (Ambion, Austin, TX) at -20°C until use. Total RNA was isolated with TRIzol reagent and treated with DNase I (TaKaRa, Shiga, Japan) at 37°C for 1 h. For standard reverse transcription-polymerase chain reaction (RT-PCR) experiments, total RNA (1 μg) was reverse transcribed using Superscript III reverse transcriptase (Invitrogen) with a 3' rapid amplification of the cDNA end (3' RACE) adapter packaged in the FirstChoice RLM-RACE kit as a primer. For 5' RACE, total RNA was modified using the FirstChoice RLM-RACE kit and reverse transcribed using Superscript III reverse transcriptase with a random decamer as a primer.

### Cloning of cDNA fragments that encode the A/B and C domains of EcR isoforms

First, we cloned cDNA fragments encoding the C domain and the C-terminal region of the A/B domain of EcR isoforms by RT-PCR. Then, we performed 5' RACE using the FirstChoice RLM-RACE kit (Ambion) to determine translation initiation site of the gene. Finally, we cloned cDNA fragments encoding the full length of the A/B domain of EcR isoforms. The obtained cDNA sequences were registered with GenBank. Detailed experimental procedures are described below.

Generally, we performed nested or semi-nested PCR to amplify a desired cDNA fragment in the RT-PCR and the 5' RACE experiments. First, PCR was performed using either *KOD-plus *polymerase (ToYoBo, Osaka, Japan) or *TaKaRa LA Taq *(TaKaRa), and nested PCR was performed using either *TaKaRa Ex Taq *(TaKaRa) or *TaKaRa LA Taq*. The amplified DNA fragments were subcloned into the plasmid vector pGEM-T easy (Promega, Madison, WI), and the nucleotide sequences were determined using ABI PRISM 3100 Genetic Analyzer (Applied Biosystems, Foster City, CA). The sequences of the degenerate/consensus primers used for the partial cDNA cloning are listed in Additional file 3, Table S3. The sequences of the primers used to amplify a cDNA encoding the full-length of the A/B domain of EcR isoforms are listed in Additional file 4, Table S2.

#### Cloning of cDNA fragments encoding the EcR C domain

To obtain the partial cDNA fragments encoding the ecdysone receptor (EcR) C domain, we performed reverse transcription polymerase chain reaction (RT-PCR) with primers designed on the basis of the highly conserved amino acid residues among the insect EcR C domain (EELCLVCGD and TCEGCKGFF for forward degenerate primers, MYMRRKCQE and VGMRAECV for reverse degenerate primers).

#### Cloning of cDNA fragments encoding the C-terminal region of the A/B domain of EcR-B1 isoform from insects and non-insect arthropods expect for Hemiptera, Hymenoptera, and Trichoptera

To obtain the cDNA fragment encoding the C-terminal region of the A/B domain of EcR-B1 isoform from insects and non-insect arthropods other than Hemiptera and Hymenoptera, we performed RT-PCR with a forward consensus primer designed on the basis of the conserved nucleotide sequence among three Coleopteran EcR-B1s (GenBank accession numbers: CAL25731, CAA72296, and BAD99297) and two Crustacean EcRs (GenBank accession numbers: AAC33432 and BAF49033). The reverse gene-specific primers were designed on the basis of the obtained cDNA sequence encoding the C domain. To determine the translation initiation site, we performed 5' RACE with gene-specific primers designed on the basis of the obtained partial cDNA encoding the A/B domain of EcR-B1 isoform.

#### Cloning of cDNA fragments encoding the C-terminal region of the A/B domain of EcR-B1 isoform from Hymenoptera

We designed an alternative forward primer on the basis of the consensus nucleotide sequence conserved among three hymenopteran insects. Briefly, we performed a genomic tBlastn search with an amino acid sequence of the *Pheidole megacephala *EcR-B1 isoform (GenBank accession number: BAE47510) against the genomic DNA sequences of *Nasonia vitripennis *and *Apis mellifera *to predict the DNA sequences encoding the A/B domain of EcR-B1 isoform. We then designed two forward consensus primers on the basis of the conserved nucleotide sequence encoding the A/B domain of hymenopteran insect EcR-B1 isoform. We simultaneously designed a reverse consensus primer on the basis of the conserved nucleotide sequence encoding the C domain of EcR. To obtain the partial cDNA fragment encoding the C-terminal region of the A/B domain of EcR-B1 isoform from Hymenoptera, we performed RT-PCR with the forward and reverse consensus primers.

#### Cloning of cDNA fragments encoding the C-terminal region of the A/B domain of EcR-B1 isoform from Hemiptera and Trichoptera

Because the forward degenerate primer described above did not work for hemipteran and trichopteran EcR-B1 gene, we performed 5' RACE with reverse gene-specific primers designed on the basis of the obtained cDNA sequence encoding the C domain of EcR.

#### Cloning of cDNA fragments encoding the C-terminal region of the A/B domain of EcR-A isoform

To obtain the cDNA fragment encoding the A/B domain of EcR-A isoform from arthropods other than Hymenoptera, we performed 5' RACE with gene-specific primers designed on the basis of the obtained partial cDNA sequence encoding the C domain of EcR.

To obtain cDNA fragment encoding EcR-A isoform from hymenopteran insects, we first designed a forward consensus primer encoding the C-terminal region of the A isoform-specific region. The forward consensus primer for hymenopteran EcR-A isoform was designed on the basis of the conserved nucleotide sequences of three hymenopteran EcR-A isoforms (GenBank accession numbers: XP_001602885, XP_394760, BAF79665, and BAE47509). We then performed RT-PCR with the forward and reverse consensus primers to obtain the partial cDNA encoding the C-terminal of the A/B domain of EcR-A isoform.

#### 5' RACE

To determine a translation initiation site of the gene, we performed 5' RACE using the FirstChoice RLM-RACE kit (Ambion) according to the manufacturer's protocol. The reverse gene-specific primers were designed based on the obtained cDNA encoding the isoform-specific region of EcR isoforms.

#### Cloning of cDNA fragments encoding the full-length of the A/B domain of EcR isoforms

To obtain a cDNA fragment encoding the full-length A/B domain of EcR isoforms, we designed a forward gene-specific primer at the 5' untranslated region (UTR) of the gene. Reverse gene specific primers were designed on the basis of the obtained cDNA corresponding to the C domain of EcR. To clone *A. mellifera *EcR isoforms, we designed a reverse gene-specific primer at the 3' UTR of the gene on the basis of the predicted full-length cDNA encoding EcR-A isoform (GenBank accession number: XM_394760).

### Sequence comparison

GenBank accession numbers of EcR-A and -B1 isoforms used for sequence comparison are listed in Additional file 1, Table S1 and Additional file 2, Table S2, respectively. The deduced amino acid sequences of the isoform-specific region of EcR-A and -B1 isoforms were aligned with the ClustalX 2.0 program [45] or MAFFT 6.0 program [46], and then manually adjusted. Prediction of potential SUMOylation sites was performed using SUMOsp 2.0 program [47]. The Sequence Logos and the consensus sequences were generated using the Geneious 4.8 program [48] to represent sequence conservation. All alignment files were supplemented in FASTA format (Additional file 5).

## Authors' contributions

TW conceived of the study, collected specimen, carried out the molecular cloning studies and the sequence alignment and drafted the manuscript. TK helped to draft the manuscript. TH advised on the manuscript. All authors read and approved the final manuscript.

## Supplementary Material

Additional file 1 **Table S1.** Taxa used in the structural comparison of the EcR-A isoform-specific region. These files can be viewed with: CLUSTAL X. Click here for file

Additional file 2 **Table S2.** Taxa used in the structural comparison of the EcR-B1 isoform-specific region. These files can be viewed with: CLUSTAL X. Click here for file

Additional file 3 **Table S3.** Degenerate/consensus primers used for cDNA cloning. These files can be viewed with: CLUSTAL X. Click here for file

Additional file 4 **Table S4. **Gene-specific primers used for cDNA cloning and the collection sites of animals. These files can be viewed with: CLUSTAL X. Click here for file

Additional file 5 **Sequence alignments of the isoform-specific regions of the EcR-A and -B1 isoforms**. Aligned sequences of the five types of the A isoform-specific regions and seven types of the B1 isoform-specific regions. These files can be viewed with: CLUSTAL X. Click here for file
