# Supplementary material for: Structural diversity and evolution of the N-terminal isoform-specific region of ecdysone receptor-A and -B1 isoforms in insects
Source: BMC Evol Biol. 2010 Feb 12;10:40. doi: 10.1186/1471-2148-10-40 (PMC2829036; doi:10.1186/1471-2148-10-40)
Supplement: Additional file 1 — Table S1. Taxa used in the structural comparison of the EcR-A isoform-specific region. These files can be viewed with: CLUSTAL X. [file 1471-2148-10-40-S1.PDF]

Table S1. Taxa used in the structural comparison of the EcR-A isoform-specific region.

	Classification	GenBank accession number	Isoform-specific region (amino acid residues)
**Non-insect arthropods**			
Daphnia magna	Subphylum Crustacea	BAF49029	1-258
Ornithodoros moubata	Subphylum Chelicerata, Order Ixodida	BAE45855	1-161
Amblyomma americanum	Subphylum Chelicerata, Order Ixodida	AAB94566	1-140
Nephila clavata	Subphylum Chelicerata, order Araneae	AB490004	1-143
Liocheles australasiae	Subphylum Chelicerata, order Scorpiones	BAF85822	1-151
**Paleoptera**			
Ephemera strigata	Order Ephemeroptera	AB490005	1-119
**Polyenoptera**			
Nemoura sp.	Order Plecoptera	AB490006	1-158
Anisolabis maritima	Order Dermaptera	AB490007	1-106
Locusta migratoria	Order Orthoptera	AAD19828	1-121
Blattella germanica	Order Dictyoptera	CAJ01677	1-150
**Paraneoptera**			
Graptopsaltria nigrofuscata	Order Hemiptera, Suborder Auchenorrhyncha	AB490009	1-145
Aphrophora pectoralis	Order Hemiptera, Suborder Auchenorrhyncha	AB490011	1-103
Bothrogonia ferruginea	Order Hemiptera, Suborder Auchenorrhyncha	AB490010	1-111
Acyrthosiphon pisum	Order Hemiptera, Suborder Sternorrhyncha	AB490013	1-168
Corythucha marmorata	Order Hemiptera, Suborder Heteroptera	AB490016	1-50
Physopelta gutta	Order Hemiptera, Suborder Heteroptera	AB490014	1-39
Pachygrontha antennata	Order Hemiptera, Suborder Heteroptera	AB490015	1-39
Liposcelis sp.	Order Psocoptera	AB490008	1-115
Pediculus humanus corporis	Order Psocoptera	EEB17490	1-114
**Endopterygota**			
Tribolium castaneum	Order Coleoptera	NP_001107650	1-151
Leptinotarsa decemlineata	Order Coleoptera	BAD99296	1-161
Apis mellifera	Order Hymenoptera, Suborder Apocrita	AB490017	1-175
Pheidole megacephala	Order Hymenoptera, Suborder Apocrita	BAE47509	1-191
Camponotus japonicus	Order Hymenoptera, Suborder Apocrita	BAF79665	1-143
Nasonia vitripennis	Order Hymenoptera, Suborder Apocrita	AB490018	1-171
Urocerus antennatus	Order Hymenoptera, Suborder Symphyta	AB490019	1-164
Panorpa pryeri	Order Mecoptera	AB490020	1-191
Stenopsyche marmorata	Order Trichoptera	AB490021	1-121
Bombyx mori	Order Lepidoptera	BAA22283	1-78
Choristoneura fumiferana	Order Lepidoptera	AAC61596	1-75
Omphisa fuscidentalis	Order Lepidoptera	ABS00248	1-76
Chilo suppressalis	Order Lepidoptera	BAC11713	1-75
Manduca sexta	Order Lepidoptera	AAB64236	1-77
Anopheles gambiae	Order Diptera, Suborder Nematocera	XP_001238299	1-247
Aedes aegypti	Order Diptera, Suborder Nematocera	P49880	1-253
Ceratitis capitata	Order Diptera, Suborder Brachycera	CAD27437	1-190
Drosophila melanoguster	Order Diptera, Suborder Brachycera	NP_724456	1-195
Drosophila yakuba	Order Diptera, Suborder Brachycera	XP_002090086	1-186
Drosophila simulans	Order Diptera, Suborder Brachycera	XP_002080273	1-196
Drosophila ananassae	Order Diptera, Suborder Brachycera	XP_001961338	1-213
Drosophila grimshawi	Order Diptera, Suborder Brachycera	XP_001985707	1-226
Drosophila mojavensis	Order Diptera, Suborder Brachycera	XP_002006838	1-258
Drosophila virilis	Order Diptera, Suborder Brachycera	XP_002049189	1-268
Drosophila persimilis	Order Diptera, Suborder Brachycera	XP_002028167	1-206
Drosophila pseudoobscura pseudoobscura	Order Diptera, Suborder Brachycera	XP_002138972	1-208
