# Supplementary material for: Structural diversity and evolution of the N-terminal isoform-specific region of ecdysone receptor-A and -B1 isoforms in insects
Source: BMC Evol Biol. 2010 Feb 12;10:40. doi: 10.1186/1471-2148-10-40 (PMC2829036; doi:10.1186/1471-2148-10-40)
Supplement: Additional file 2 — Table S2. Taxa used in the structural comparison of the EcR-B1 isoform-specific region. These files can be viewed with: CLUSTAL X. [file 1471-2148-10-40-S2.PDF]

Table S2. Taxa used in the structural comparison of the EcR-B1 isoform-specific region.

	Classification	GenBank accession number	Isoform-specific region (amino acid residues)
**Non-insect arthropods**			
Celuca pugilator	Subphylum Crustacea	AAC33432	1-121
Daphnia magna	Subphylum Crustacea	BAF49033	1-196
Thereuopoda clunifera	Subphylum Myriapoda	AB490023	1-63
Nephila clavata	Subphylum Chelicerata, order Araneae	AB490022	1-123
**Apterygota**			
Thermobia domestica	Order Thysanura	AB490024	1-73
Ctenolepisma villosa	Order Thysanura	AB536931	1-73
**Paleoptera**			
Ephemera strigata	Order Ephemeroptera	AB490025	1-81
Sympetrum infuscatum	Order Odonata	AB490026	1-103
**Polyenoptera**			
Nemoura sp.	Order Plecoptera	AB490027	1-84
Anisolabis maritima	Order Dermaptera	AB490028	1-91
Gryllus bimaculatus	Order Orthoptera	AB536932	1-132
Achetus domesticus	Order Orthoptera	AB490030	1-136
Locusta migratoria	Order Orthoptera	AB490029	1-101
Tenodera angustipennis	Order Mantodea	AB490033	1-97
Reticulitermes speratus	Order Isoptera	AB490032	1-131
Periplaneta fuliginosa	Order Blattaria	AB490031	1-113
**Paraneoptera**			
Graptopsaltria nigrofuscata	Order Hemiptera, Suborder Auchenorrhyncha	AB490037	1-102
Oncotympana maculaticollis	Order Hemiptera, Suborder Auchenorrhyncha	AB536933	1-102
Aphrophora pectoralis	Order Hemiptera, Suborder Auchenorrhyncha	AB490039	1-101
Bothrogonia ferruginea	Order Hemiptera, Suborder Auchenorrhyncha	AB490038	1-98
Acyrthosiphon pisum	Order Hemiptera, Suborder Sternorrhyncha	AB490041	1-106
Physopelta gutta	Order Hemiptera, Suborder Heteroptera	AB490042	1-48
Pachygrontha antennata	Order Hemiptera, Suborder Heteroptera	AB490044	1-47
Orius strigicollis	Order Hemiptera, Suborder Heteroptera	AB490043	1-52
Corythucha marmorata	Order Hemiptera, Suborder Heteroptera	AB490045	1-94
Appasus japonicus	Order Hemiptera, Suborder Heteroptera	AB536934	1-53
Franklinothrips vespiformis	Order Thysanoptera	AB490035	1-102
Liposcelis sp.	Order Psocoptera	AB490034	1-96
**Endopterygota**			
Anthonomus grandis	Order Coleoptera	ACK57879	1-97
Leptinotarsa decemlineata	Order Coleoptera	BAD99297	1-87
Tenebrio molitor	Order Coleoptera	CAA72296	1-94
Tribolium castaneum	Order Coleoptera	CAL25731	1-89
Pseudoxenos iwatai	Order Strepsiptera	AB490049	1-115
Hagenomyia micans	Order Neuroptera	AB490048	1-117
Protohermes grandis	Order Megaloptera	AB490047	1-128
Inocellia japonica	Order Raphidioptera	AB490046	1-94
Apis mellifera	Order Hymenoptera, Suborder Apocrita	AB490050	1-106
Bombus hypocrita	Order Hymenoptera, Suborder Apocrita	AB490051	1-106
Xylocopa appendiculata	Order Hymenoptera, Suborder Apocrita	AB490052	1-106
Osmia cornifrons	Order Hymenoptera, Suborder Apocrita	AB490053	1-106
Coelioxys fenestrata	Order Hymenoptera, Suborder Apocrita	AB490054	1-106
Ammophila infesta	Order Hymenoptera, Suborder Apocrita	AB490055	1-133
Vespa mandarinia	Order Hymenoptera, Suborder Apocrita	AB490056	1-181
Anterhynchium flavomarginatum	Order Hymenoptera, Suborder Apocrita	AB490057	1-147
Scolia oculata	Order Hymenoptera, Suborder Apocrita	AB490059	1-143
Campsomeris schulthessi	Order Hymenoptera, Suborder Apocrita	AB490060	1-118
Pheidole megacephala	Order Hymenoptera, Suborder Apocrita	BAE47510	1-99
Anospilus sp.	Order Hymenoptera, Suborder Apocrita	AB490058	1-118
Amblyjoppa sp.	Order Hymenoptera, Suborder Apocrita	AB490061	1-141
Eretmocerus eremicus	Order Hymenoptera, Suborder Apocrita	AB490063	1-161
Nasonia vitripennis	Order Hymenoptera, Suborder Apocrita	AB490062	1-110
Urocerus antennatus	Order Hymenoptera, Suborder Symphyta	AB490064	1-102
Cimbex femoratus	Order Hymenoptera, Suborder Symphyta	AB490065	1-135
Arge similis	Order Hymenoptera, Suborder Symphyta	AB490066	1-151
Allantus luctifer	Order Hymenoptera, Suborder Symphyta	AB490068	1-133
Tenthredo fagi	Order Hymenoptera, Suborder Symphyta	AB490067	1-131
Panorpa pryeri	Order Mecoptera	AB490069	1-123
Stenopsych marmorata	Order Trichoptera	AB490070	1-113
Bombyx mori	Order Lepidoptera	AAA87341	1-172
Spodoptera frugiperda	Order Lepidoptera	AAM54494	1-152
Omphisa fuscidentalis	Order Lepidoptera	ABS00249	1-106
Chilo suppressalis	Order Lepidoptera	BAC11714	1-107
Manduca sexta	Order Lepidoptera	P49883	1-112
Aedes albopictus	Order Diptera, Suborder Nematocera	AAA87394	1-155
Calliphora vicina	Order Diptera, Suborder Brachycera	AAG46050	1-279
Lucilia cuprina	Order Diptera, Suborder Brachycera	AAB81130	1-264
Ceratitis capitata	Order Diptera, Suborder Brachycera	CAA11907	1-211
Drosophila melanogaster	Order Diptera, Suborder Brachycera	NP_724460	1-227
Drosophila simulans	Order Diptera, Suborder Brachycera	XP_002080271	1-225
Drosophila yakuba	Order Diptera, Suborder Brachycera	XP_002090088	1-224
Drosophila ananassae	Order Diptera, Suborder Brachycera	XP_001961340	1-235
Drosophila grimshawi	Order Diptera, Suborder Brachycera	XP_001985709	1-262
Drosophila mojavensis	Order Diptera, Suborder Brachycera	XP_002006840	1-248
Drosophila virilis	Order Diptera, Suborder Brachycera	XP_002049186	1-260
Drosophila persimilis	Order Diptera, Suborder Brachycera	XP_002028169	1-255
Drosophila pseudoobscura pseudoobscura	Order Diptera, Suborder Brachycera	XP_002138971	1-256
