# Supplementary material for: Structural diversity and evolution of the N-terminal isoform-specific region of ecdysone receptor-A and -B1 isoforms in insects
Source: BMC Evol Biol. 2010 Feb 12;10:40. doi: 10.1186/1471-2148-10-40 (PMC2829036; doi:10.1186/1471-2148-10-40)
Supplement: Additional file 3 — Table S3. Degenerate/consensus primers used for cDNA cloning. These files can be viewed with: CLUSTAL X. [file 1471-2148-10-40-S3.PDF]

Table S3. Degenerate/consensus primers used for cDNA cloning.

**Degenerate/consensus primers for the EcR-C domain**	
Forward degenerate primer_1 (EELCLVCGD)	TCACCTGYGARGGHTGYAARGG
Forward degenerate primer_2 (TCEGCKGFF)	ACSTGYGARGGHTGYAAAGGKTTCT
Reverse degenerate primer_1 (VGMRAECV)	CTTGRCACTTYCKYCKCATRTACAT
Reverse degenerate primer_1 (MYMRRKCQE)	ARCATCATIACYTCISWTGADCADGC
Reverse consensus primer for Hymenoptera EcR-C domain	CGCATGTACATGTCGATCTC
**Forward consensus primers for the EcR-A/B domain**	
Forward consensus primer for EcR-B1 isoform	GATCTCGAGTTCTGGACCTGGA
Forward consensus primer for Hymenoptera EcR-A isoform	ATGAGCACCAGCAGCTACGATCC
Forward consensus primer for Hymenoptera EcR-B1 isoform_1	CCCGGAAGTATCGAGCAGC
Forward consensus primer for Hymenoptera EcR-B1 isoform_2	CCATGGAAGATTTACAGCTTTGGGA
