# Supplementary material for: Structural diversity and evolution of the N-terminal isoform-specific region of ecdysone receptor-A and -B1 isoforms in insects
Source: BMC Evol Biol. 2010 Feb 12;10:40. doi: 10.1186/1471-2148-10-40 (PMC2829036; doi:10.1186/1471-2148-10-40)
Supplement: Additional file 4 — Table S4. Gene-specific primers used for cDNA cloning and the collection sites of animals. These files can be viewed with: CLUSTAL X. [file 1471-2148-10-40-S4.PDF]

**Table S4. Gene-specific primers used for cDNA cloning and the collection sites of animals.**

| | A isoform 5' UTR primer | B1 isoform 5' UTR primer | Reverse outer primer | Reverse inner primer | Collection sites |
|---|---|---|---|---|---|
| *Nephila clavata* | GACCTCAAATGGATACGCTGATTC | GGGCTGTGAGAAGACAAAGCA | GGCATTTTCTCCTCATGTACATGTC | GCCATATTTGCACTGGTACACTG | Tokyo, Japan |
| *Thereuopoda clunifera* | - | CTGGGAAAGTGTGTTGAACTC | CTTCTTCAACCTGCATTCTTGAC | CTCCTCATGTACATGTCAATTTCAC | Misaki Marine Biological Station, Misaki, Kanagawa Pref., Japan |
| *Thermobia domestica* | - | CCGAGTTTCTGTGGTGAATTATTG | CCGGCATTCTTGACATTTTCTCCTC | GGCCGTATTTGCATTGATATACTG | Purchased form the local pet store |
| *Ctenolepisma villosa* | - | GGAAGTTGCTCACGTTGTTGC | CCGGCATTCTTGACATTTTCTCCTC | - | Tokyo, Japan |
| *Ephemera strigata* | CATACTGCTTTGGAACTTGATTACTGC | ATATTGGTGCTTCGTTAGTGTGCTCGTG | CACTTACGCCGCATATACATGTC | GCCGCATATACATGTCAATTTCACAG | Lake Shikotsu, Hokkaido, Japan |
| *Sympetrum infuscatum* | - | GTTCCGAAAGTGAAGTTCATGTC | CGACATTCCTGACATTTTCTCCTC | CTCCTCATGTACATGTCAATCTCAC | Tokyo, Japan |
| *Nemoura sp.* | GTGCGAAAACAAAGTGGTTTTAGC | CCGAAATCAGTCAAAAAGCAGTGATTAG | CGTCGGAAGAAACCTTTGCATC | CGCAAGTGAGAGCATTGTAGTG | Mt. Teine, Hokaido, Japan |
| *Anisolabis maritima* | CAACAAGGACAACAAATTTGGTG | CCTCATTGGGTACGTAGATAATTAG | GTCGACACTCTTGACATTTCCTTC | CCTTCGCATATACATGTCAATCTC | Tokyo, Japan |
| *Locusta migratoria* | - | CTGTTGTGTGCGGTGTTTTAAACG | GCCATATTTGCACTGGTACACG | GCAGGTGAGAGCATTGTAGTG | Chiba, Japan |
| *Gryllus bimaculatus* | - | TGGCGCCCAATATGATCTTGC | CCTACACTCTTGGCACTTC | CACAATTGTTACCATATTTGCACTG | Purchased form the local pet store |
| *Acheta domesticus* | - | CGTGTGGTGATGCTGGTGAC | CCTACACTCTTGGCACTTC | CACAATTGTTACCATATTTGCACTG | Purchased form the local pet store |
| *Periplaneta fuliginosa* | - | GACAGTTCAATCTCCTGATGTTTGTTC | GTCGCATGTACATGTCGATTTC | CTTGCACTGATACACTGCATTC | Tokyo, Japan |
| *Reticulitermes speratus* | - | CAATCAGTGACGTTGTGTAACC | CCGTCGCATGTACATGTCAATC | CTCACAGTTGTTCCCATACTTG | Tokyo, Japan |
| *Tenodera angustipennis* | - | CTGCGGCTTAAACTCTCAAGTC | CCGACACTCTTGGCACTTC | GCACTGGTAGACTGCATTC | Tokyo, Japan |
| *Liposcelis sp.* | CATACCGCATTCGTAGTAGC | GTGGTTAGTGAAGATTGTTTTTCCG | GACACTTTCGCCTCATGTACATATC | CCCCATATTTACACTGATACACTG | Tokyo, Japan |
| *Franklinothrips vespiformis* | - | GTGACCAGTCTCTCGTGTG | CCATATTTGCACTGGTACACAGC | TTTGCACCCCTCACAGGTAAG | Purchased form Arysta LifeScience |
| *Graptopsaltria nigrofuscata* | GCTGTTGAGAAGAGGACACTAGC | GCCAAAAGTGGTATTAACGTTC | CACTGAGACATTTCTTAAGCCTAC | GCCTCATGTACATGTCTATTTCAC | Tokyo, Japan |
| *Oncotympana maculaticollis* | - | GAGTGTCATTCCCAGGGTTATTAG | CACTGAGACATTTCTTAAGCCTAC | GCCTCATGTACATGTCTATTTCAC | Tokyo, Japan |
| *Bothrogonia ferruginea* | GTGCTGAGGTGTTTACTACTGG | CGTGACATTCCAGGCCTAGC | CATGTCGATCTCGCAGTTTGTG | GCCATACTTGCACTGGTAGAC | Tokyo, Japan |
| *Aphrophora pectoralis* | GTCGCGTGTGTGGTGTTAC | CGCGAGGTATTGTGGAATCAAAAC | CTTCAGTCTACACTCTTGACATTTCC | GACATTTCCTCCTCATGTACATGTC | Lake Shikotsu, Hokkaido, Japan |
| *Acyrthosiphon pisum* | CAACGTGGCGACGTTCGAG | CCCGGTTATCTGGTCATACG | CATGTCAGCGCGTTGTAATGG | GTCGGTTGTTCAGCACGTTG | Kindly provided from K. Nishikori |
| *Physopelta gutta* | GGTGGTGACAGACCCATTC | GTTCAGTGATCGTAGTGTACAAGTG | CTACACTCTTGACATTTCCGTCTC | CTCATGTACATGTCTATCTCACAG | Tokyo, Japan |
| *Orius strigicollis* | - | GTGGGATCAACACGACTCAAC | CATGTCGATTTCGCAATTGTTTCC | - | Purchased form Arysta LifeScience |
| *Pachygrontha antennata* | GATCGACACAACGCGAATTGG | CCGTCTGTGAGTGTTGTGTTC | GAGCCTACACTCCTGACATTTC | CATGTCGATCTCGCAGTTGTTC | Tamagawa river, Kanagawa, Japan |
| *Corythucha marmorata* | GTTTTTCACGCTGAAATTCCTGGAAG | GTGTATCGCTTGCGACAGG | CCTCATGTACATGTCTATTTCACAG | CTGGTAGACGGCATTTTTCGTG | Tamagawa river, Kanagawa, Japan |
| *Appasus japonicus* | - | GCACACTCAGGAGGAGCATC | GCGGCATTCCTGGCACTTG | - | Kyoto, Japan |
| *Inocellia japonica* | - | GTGTCGAAAGTGTGCGATGTTG | CCCATACTTGCACTGGTATACTG | CTTCTTCTGAAGAAACCTTTACATCC | Chiba, Japan |
| *Protohermes grandis* | - | GAGTAACGAACGACTTGTAAATCC | CCTGCACTCTTGGCACTTC | CTCCGCATGTACATGTCCATC | Sagami River, Kanagawa. Japan |
| *Hagenomyia micans* | - | GTTGTATTTTAGTCGGTGTGATGTTTTTAACG | GTCGACATTCTTGACATTTACG | GGCATTTTTTGTTATACTCCGTC | Tokyo, Japan |
| *Pseudoxenos iwatai* | - | CAGTGATGTTACTTCAGAAGAAAAAGTG | CATTCTTGACACTTCCGTCTC | CAGCATTTTTAGTTACACTTCGTCG | Misaki Marine Biological Station, Misaki, Kanagawa, Japan |
| *Apis mellifera* | GTGTCGTGTTGGCAGCCACTC | CGTTGCACTGAGAGGAAAGGAAAG | GACGTCACCTGTGTCTACTAATC | CTCGCGATGAAACAACACTATCAG | Purchased form Kumagawa bee farm |
| *Bombus hypocrita* | - | CTGGCGAGATGCTGCAGACGGTG | CGCAATTGTTCCCGTATTTACACTG | GTATACCGCGTTCTTGGTGATG | Mt. Teine, Hokaido, Japan |
| *Xylocopa appendiculata* | - | CGACGAACGAATTATCGAGGTCAAC | CGCATGTACATGTCGATCTC | - | Tokyo, Japan |
| *Osmia cornifrons* | - | CGAACGAATTATCGAGGTCAACG | CGCATGTACATGTCGATCTC | - | Purchased form Mason Bee Research Institute |
| *Coelioxys fenestrata* | - | CGACGAACGAATTATCGAGGTCAAC | CGCATGTACATGTCGATCTC | - | Tokyo, Japan |
| *Ammophila infesta* | - | CGGAGAAGGTGTAACACAG | AGTTGTTCCCGTACTTGCACTG | ATAGACCGCGTTCTTCGTGATG | Mt. Teine, Hokaido, Japan |
| *Vespa mandarinia* | - | GGATAAGATCAAAGAGGAACAGG | CGCATGTACATGTCGATCTC | - | Tokyo, Japan |
| *Anterhynchium flavomarginatum* | - | CTTGTGCGATATAAACAAACGAGG | CGCATGTACATGTCGATCTC | - | Misaki Marine Biological Station, Misaki, Kanagawa, Japan |
| *Anospilus sp.* | - | GTGTATGTGTGTATGTGTGTTAGAAGAG | CGCATGTACATGTCGATCTC | - | Mt. Teine, Hokaido, Japan |
| *Scolia oculata* | - | GTTCGTGGAAATCGAAGTGG | CAATCTACACTCCTGGCACTTG | CAATTGTTGCCGTACTTGCATTG | Tokyo, Japan |
| *Campsomeris schulthessi* | - | CTGTATCGCGTTGCAAAGGAG | CTCCTCATGTACATGTCAATCTCAC | - | Tokyo, Japan |
| *Amblyjoppa sp.* | - | CGTAAGGGAGTGTGAATTGTC | GGAAGAAGCCCTTGCAC | - | Mt. Teine, Hokaido, Japan |
| *Nasonia vitripennis* | CCATCAGCGTTGAAGCCAGTC | GCGCGTACGAGCAGTTTAG | CTTCTTCTCCTTGCGCTTGAC | GCCTCATGTACATGTCGATCTC | Purchased form Nosan corporation |
| *Eretmocerus eremicus* | - | CGACGGCATCAGGGTGAAC | CGCATGTACATGTCGATCTC | - | Purchased form Arysta LifeScience |
| *Urocerus antennatus* | GTCACGGTGGCCAGCTTAGTG | GGGTTCCTTCTCTTTTGCAC | CTCGCAGTTATTGCCGTACTTG | ACTGGTACACGGCGTTCTTC | Sapporo, Hokkaido, Japan |
| *Tenthredo fagi* | - | CTAACTCATCACAGTACTTACCAC | CGCATGTACATGTCGATCTC | - | Mt. Teine, Hokaido, Japan |
| *Allantus luctifer* | - | GTCATTGAAACACTGTGACAGTGC | CGCATGTACATGTCGATCTC | - | Tokyo, Japan |
| *Arge similis* | - | GTGCGACGGTAAAAGGTGAATG | CCGACACTCCTGACACTTTC | ATGTCGATCTCGCAATTGTTTCC | Tokyo, Japan |
| *Cimbex femoratus* | - | GGAACTACTAGAGTACGTTATAGTG | CTGCACTCCTGACATTTTCTTCG | CATGTACATGTCGATCTCGCAG | Sapporo, Hokkaido, Japan |
| *Panorpa pryeri* | GATCAGAGACTTGTCAATTTATTGTCG | GCTTCTTCGCTGATTCTGATCG | AGTCTGCATTCCTGACACTTCC | CTGACACTTCCTTCTCATATACATATC | Mt. Teine, Hokaido, Japan |
| *Stenopsyche marmorata* | GCCGCTGGAGCTGAC | GTGCGCACGCGTTCGCAC | CATGTCCATCTCGCATTCGTG | GCATTTTTGGTGACGCTCCTTC | Ara river, Saitama, Japan |
